# Acute Exposure to Permethrin Modulates Behavioral Functions, Redox, and Bioenergetics Parameters and Induces DNA Damage and Cell Death in Larval Zebrafish

**DOI:** 10.1155/2019/9149203

**Published:** 2019-11-11

**Authors:** Mauro Eugênio Medina Nunes, Lucia Emanueli Schimith, Dennis Guilherme da Costa-Silva, Andressa Rubim Lopes, Luana Paganotto Leandro, Illana Kemmerich Martins, Renata Siqueira de Mello, Diane Duarte Hartmann, Nelson Rodrigues de Carvalho, Pamela Carvalho da Rosa, Rafael Trevisan, Richard Thomas Di Giulio, Thaís Posser, Jeferson Luis Franco

**Affiliations:** ^1^Oxidative Stress and Cell Signaling Research Group, Interdisciplinary Center for Biotechnology Research-CIPBIOTEC, Campus São Gabriel, Federal University of Pampa, São Gabriel, RS 97300-000, Brazil; ^2^Department of Molecular Biology and Biochemistry, Graduate Program in Biological Sciences: Toxicological Biochemistry, Federal University of Santa Maria, 1000 Roraima Avenue, Santa Maria, RS 97105-900, Brazil; ^3^Nicholas School of the Environment, Duke University, Durham, NC 27708, USA

## Abstract

Permethrin (PM) is a synthetic pyrethroid insecticide widely used as domestic repellent. Damage effects to nontarget organisms have been reported, particularly in the early stages of development. Studies indicate redox unbalance as secondary PM effect. Therefore, our goal was to investigate the acute PM effects on larval zebrafish. Larvae (6 days postfertilization) were exposed to PM (25–600 *μ*g/L) during 24 hours, and 50% lethal concentration was estimated. For subsequent assays, the sublethal PM concentrations of 25 and 50 *μ*g/L were used. PM increased anxiety-like behaviors according to the Novel Tank and Light-Dark tests. At the molecular level, PM induced increased ROS, which may be related to the increased lipid peroxidation, DNA damage, and apoptosis detected in PM-exposed organisms. In parallel, upregulation of the antioxidant system was detected after PM exposure, with increased superoxide dismutase, glutathione S-transferase and glutathione reductase activities, and thiol levels. The increased of Nrf2 target genes and the activation of an electrophile response element-driven reporter Tg(*EPRE*:LUC-EGFP) suggest that the Nrf2 pathway can mediate a fast response to PM, leading to antioxidant amplification. By using high-resolution respirometry, we found that exposure to PM decreased the oxygen consumption in all respiratory stages, disrupting the oxidative phosphorylation and inhibiting the electron transfer system, leading to decrease in bioenergetics capacity. In addition, PM led to increases of residual oxygen consumption and changes in substrate control ratio. Glucose metabolism seems to be affected by PM, with increased lactate dehydrogenase and decreased citrate synthase activities. Taken together, our results demonstrated the adverse effects of acute sublethal PM concentrations during larval development in zebrafish, causing apparent mitochondrial dysfunction, indicating a potential mechanism to redox unbalance and oxidative stress, which may be linked to the detected cell death and alterations in normal behavior patterns caused by acute PM exposure.

## 1. Introduction

Permethrin (PM; 3-phenoxybenzyl-(1R,S)-cis,trans-3-(2,2-dichlorovinyl)-2,2-dimethylcyclopropane carboxylate) is one of the most frequently used Type I synthetic pyrethroids (SP) [1]. The main molecular targets of SP are the voltage-dependent sodium channels (VDSC) to which they bind and delay the inactivation of sodium channels, resulting in neurotoxicity effects and ultimately death [2]. This mode of action is common to all organisms exposed to SP [2, 3].

PM is used in a range of insecticide formulas to control pests in residential and agricultural areas due to its high efficiency and low mammalian toxicity [4]. However, several studies have shown that PM can cause a variety of side effects in nontarget organisms, including teratogenicity, cardiotoxicity, endocrine dysfunction, hepatotoxicity, and cytotoxicity both in vertebrates and invertebrates (for a review see [5]). Besides that, studies have found detectable levels of the SP metabolite 3-phenoxybenzoic acid in children and pregnant women [6–10], though little is known on the mechanisms by which SP induce toxicity during the early stages of life.

In general, developing organisms are most vulnerable to toxic effects of SPs [11] and epidemiologic studies demonstrated that exposure to pesticides during pregnancy and*/*or early childhood is associated with neurodevelopmental outcomes in children [12], which can change functional neurodevelopmental endpoints such as behavior [13]. This higher sensitivity of developing organisms has been associated with cellular redox unbalance, which can impair normal development [14, 15]. Additionally, other evidences have indicated oxidative stress as a secondary mode of action of PM due to changes in redox biomarkers in different animal models, including DNA, lipid and protein damage, modulation of the antioxidant system [5, 16–18], and activation or repression of transcription factors regulating proinflammatory, anti-inflammatory, and apoptotic responses [19, 20].

Oxidative phosphorylation (OXPHOS) is one of the metabolic pathways sensitive to environmental toxicants [21]. Mitochondrial dysfunction can lead to increase in the partial reduction of oxygen by electrons leaking from the mitochondrial electron transport chain (ETC), generating an increase in reactive oxygen species (ROS) steady-state levels and, potentially, oxidative stress [22]. Besides that, the mitochondrial ATP production exerts several functions in cell metabolism, such as energy metabolism, ROS production and elimination, and cell death [23, 24]. Thereby, the mitochondrial OXPHOS and the ETC can be the possible targets of PM toxicity and influence cellular fate and function through redox unbalance.

Alternative experimental models have arisen as important tools in toxicology, providing valuable information about the mechanisms of toxicity of several chemical molecules [25]. Among them, zebrafish (*Danio rerio*) appears as an important vertebrate model [26, 27], with substantial genetic and physiological homology in relation to humans and easily genetically manipulated [28]. These advantages have led to the use of the zebrafish model in drug discovery, toxicological screening, and developmental toxicology [29].

The present study sought to determine toxicological effects of acute PM exposure and the relationship between changes in behavior patterns, redox parameters, mitochondrial function, and cell death in zebrafish larvae acutely exposed to sublethal concentrations of PM.

## 2. Materials and Methods

### 2.1. Chemicals and Reagents

Except for the lactate dehydrogenase activity kit (Labtest, Lagoa Santa, MG, Brazil), SYBR Safe DNA gel stain (Life Technologies, Carlsbad, CA, USA), and anti-*γ*-GCS, HO-1, and NQO-1 primary antibodies (Santa Cruz Biotechnology Inc., Dallas, TX, USA), all other chemicals were purchased from Sigma-Aldrich (São Paulo, SP, Brazil).

### 2.2. Zebrafish Maintenance and Reproduction

Adult *Danio rerio* (wild type) were obtained from a local commercial supplier and maintained in a recirculating aquatic system (Zebtec®) under appropriate water conditions (pH 7.2 ± 0.5, 400 ± 50 *μ*S conductivity, 28 ± 1°C temperature, and dissolved oxygen equal or above 95% saturation) with a 14 h : 10 h (light : dark) photoperiod. Ammonia, nitrite, and nitrate values were kept lower than 0.2 ppm, 0.05 ppm, and 0.05 ppm, respectively. This water was used in the preparation of test solutions of all assays performed. The fish were fed on commercial flocked fish food and supplemented with brine shrimp (*Artemia salina*). The experimental protocols used in this work were approved by the local ethics committee (CEUA, Unipampa: protocol 003-2016).

Experiments also used the transgenic lines Tg(*EPRE*:LUC-EGFP) (a generous gift from Dr. Michael Carvan, University of Wisconsin, WI, USA) [30] and Tg[(*HSP70*:EGFP)*_unspecified*] (a generous gift from Dr. Michael Hahn, Woods Hole Oceanographic, MA, USA, and Dr. John Y. Kuwada, University of Michigan, MI, USA) [31, 32]. These animals were maintained in a recirculating AHAB system (Aquatic Habitats Inc., Apopka, FL, USA) on a 14 : 10 h light/dark cycle. Water quality was maintained at 27-29°C, pH 7.0-8.0, using 60 ppm artificial seawater (Instant Ocean, Foster & Smith, Rhinelander, WI, USA). The fish were fed on Zeigler's Adult Zebrafish Complete Diet (Aquatic Habitats Inc.) and supplemented with brine shrimp. All procedures were approved by the Institutional Animal Care and Use Committee of the Duke University (IACUC protocol: A139-16-06).

Male and female adult fish (6–12 months) with an optimal ratio of 2 : 1 were placed in pairs overnight. The reproduction was induced by the light irritation on the next morning. Fertilized eggs were collected, washed with fish system water for several times, and incubated in system water in a BOD (Biochemical Oxygen Demand) incubator at 28°C.

### 2.3. PM Exposure and Survival Rate

The stock solution of permethrin (PM, 98.3%, a mixture of isomers) was prepared in ethanol and stored at -20°C in nominal concentrations of 25, 50, 75, 100, 200, 300, and 600 mg/L. Toxicological assays were based on the OECD guidelines for the testing of chemicals 210, Fish, Early-life Stage Toxicity Test [33], with some modifications. Briefly, a number of 50 larvae zebrafish (per group) with 6 days postfertilization (dpf) were selected by simple randomization for acute exposure by immersion to PM during 24 h in 50 mL plastic tubes (falcon tubes), in a total of 6 replicates for each independent experiments (totalizing 300 larvae/concentration/experiment). For LC_50_ (defined as the concentration causing 50% of mortality in the exposed animals) and other experiments, larvae were exposed to nominal concentrations of 0, 25, 50, 75, 100, 200, 300, and 600 *μ*g/L of PM during 24 h. The exposure solution was diluted in water of the maintenance system (WMS), and the final concentration of ethanol in each treatment, including the control group (CTL), was 0.01% (*v*/*v*). The sublethal concentrations and sample size were based on dose effects demonstrated in experiments performed by Demicco et al. [34] and Yang et al. [18]. For the consequent analyses, the concentrations that will not increase mortality and did not present teratogenicity effects according to the literature will be chosen [18, 34], and the larvae were exposed for 24 h in falcon tubes (50 larvae per tube; 100–600 larvae per group, 2–12 replicates of 50 larvae in total) contacting 50 mL of PM solutions.

### 2.4. Behavior Assessment

The larval behavior assays were adapted to the larval developmental stage and followed the adult methods described in the previous article, published in *Molecular Neurobiology* [35]. Briefly, the behavioral tests were performed after the time exposure between 1:00 and 5:00 pm. After time of exposure, a number of 35–50 larvae were selected by simple randomization and individually placed in each well of a 24-well cell culture plate (hereafter called apparatus), filled with WMS (2 mL, 27 ± 1°C), and the behavioral activities of zebrafish were recorded for a single session of 300 seconds. The experimental procedures were performed on a stable surface with all environmental distractions kept to a minimum. For swimming location and determination of behavioral parameters, we followed the same methods described by Nunes et al. [35].

#### 2.4.1. Open Field Test

Locomotors and exploratory activities were analyzed in the Open Field test. The swimming pattern behavior was analyzed as described elsewhere [36]. The behavioral activities were recorded after 300 seconds of habituation. The apparatus was virtually divided into two circular sections (central and periphery) to assess the spatial exploration by the following endpoints: total time and average time spent per visit in the central zone (s), which were used in measuring the fear/anxiety-related behaviors. Total distance traveled (m), absolute turn angle (°), and total immobility time (s) were used to measure locomotors and motor patterns.

#### 2.4.2. Novel Tank Test

The exploratory behavior followed the established protocols using zebrafish larvae [36, 37], which were originally adapted from adult behavior tests [35, 38, 39]. The behavioral activities in the Novel Tank test were recorded without habituation time, which may reflect a direct response to novelty stress in contrast to the Open Field test. The apparatus was virtually divided into two circular sections (central and periphery areas) to assess the spatial exploration by the following endpoints: total time and average time spent per visit in the periphery (s), which were used to estimate the fear/anxiety-related behaviors. Total distance traveled (m) and total time immobility (s) were used to measure locomotors and motor patterns.

#### 2.4.3. Light-Dark Preference Test

This test was adapted from light/dark preference behavioral assays carried out with adult [35, 40] and larval zebrafish [41]. The surface of the apparatus was physically divided into two areas (black and white) of equal size, using black or white opaque tapes and no physical barrier between them. Each animal was placed initially in the lit (white) area, and the number of entries into the dark area, total time spent (s) in the lit area (s), latency to enter the dark area (s), and the number of risk assessment episodes were measured. Risk assessments were defined as a partial entry in the dark area followed by a fast return to the lit area.

### 2.5. Measurement of ROS Steady-State Levels

The ROS steady-state levels were measured using the fluorescent dye 2,7-dichlorofluorescein-diacetate (DCFDA) [42], following methods described in the previous article, published in *Molecular Neurobiology* [34]. At the end of the exposure, twenty-five larvae were pooled per sample (*n* = 6 per group).

### 2.6. Lipid Peroxidation Estimation Assay

Lipid peroxidation was estimated by thiobarbituric acid reactive substance (TBARS) assay [43], following methods described in the previous article, published in *Molecular Neurobiology* [34]. At the end of the exposure, twenty-five larvae were pooled per sample (*n* = 6 per group).

### 2.7. Antioxidant Enzyme Activity

Antioxidant enzyme measurements were performed using six independent experiments per group (*n* = 6), and twenty-five larvae were pooled per sample, following methods described in the previous article, published in *Molecular Neurobiology* [34].

Catalase (CAT) activity was assessed by measuring the rate of decrease in H_2_O_2_ absorbance at 240 nm [44]. The specific activity was determined in a cuvette reader using the extinction coefficient of 40 M/cm and expressed as *μ*mol/min/mg of protein.

Superoxide dismutase (SOD) activity was analyzed through the inhibition capacity of quercetin oxidation (406 nm) by superoxide radical in the presence of N,N,N′,N′-tetramethylethane-1,2-diamine (TEMED) at pH 10 [45]. The SOD activity is expressed in units SOD/mg of total protein, where 1 unit is the amount of SOD required to give 50% maximal inhibition of the initial rate of quercetin reduction.

Glutathione peroxidase activity (GPx, selenium-dependent) was measured following the rate of NADPH oxidation at 340 nm, in the presence of GSH, glutathione reductase (GR), and H_2_O_2_ [46].

The GR activity was measured following the rate of NADPH oxidation at 340 nm by reduction of GSSG to GSH. The assay mixture consisted of potassium phosphate buffer (100 mM, pH 7.0), 0.15 mM NADPH, and 1 mM GSSG. The specific activity was determined using the extinction coefficient of 6.22 mM/cm and expressed as nmol/min/mg of protein.

Glutathione S-transferase (GST) activity was measured following conjugation of 1-chloro-2,4-dinitrobenzene (CDNB) with GSH at 340 nm [47].

### 2.8. Determination of Thiol Levels

Ten larvae were pooled per sample (*n* = 6 per group), following methods described in the previous article, published in *Molecular Neurobiology* [34]. The fluorescence related to the thiol levels (nonprotein thiols) was read at 350 nm (ex) and 420 (em) [48].

### 2.9. Western Blotting Analysis

Western blotting was performed according to a previous protocol from our group using zebrafish [49], with minor modifications. Fifty larvae were homogenized per sample (*n* = 4 per group) in Tris NaF buffer (50 mM Tris pH 7.0 containing 1 mM EDTA, 0.1 mM phenylmethyl sulfonyl fluoride, 20 mM Na_3_VO_4_, 100 mM sodium fluoride, and protease inhibitor cocktail), and 10 *μ*L of sample was taken out for protein analysis prior to incubation with 4% SDS stop solution (4% SDS, 50 mM Tris, 100 mM EDTA, pH 6.8). Samples were further mixed with 25% glycerol and 8% *β*-mercaptoethanol. Proteins were separated by SDS-PAGE using 12% gels and then electrotransferred to nitrocellulose membranes for approximately 3 hours. The membranes were blocked with 5% skimmed milk for 1 hour, washed in Tris-buffered saline with Tween-20 (100 mM Tris-HCl, pH 7.5, 0.9% NaCl, and 0.1% Tween-20), and incubated overnight at 4°C with the following rabbit primary antibodies: *γ*-GCS polyclonal antibody (rabbit anti-human; 1 : 1,000 dilution; cat. no. sc-22755; Santa Cruz Biotechnology), HO-1 (rabbit anti-human; 1 : 1,000 dilution; cat. no. sc-10789; Santa Cruz Biotechnology), NQO-1 (rabbit anti-human; 1 : 1,000 dilution; cat. no. sc-16464; Santa Cruz Biotechnology), and anti-*β*-actin polyclonal antibody (rabbit anti-human; 1 : 1,000 dilution; cat. no. A5060; Sigma-Aldrich). Subsequently, membranes were washed in Tris-buffered saline with Tween-20 and incubated for 1 hour at room temperature with horseradish peroxidase-linked anti-IgG (1 : 10000) secondary specific antibodies. The immunoblots were visualized on the IS4000MM Pro Bruker imaging system using the ECL detection reagent, and the band density was quantified using the Scion Image® software.

### 2.10. Assessment of Antioxidant Response *In Vivo* Using Zebrafish Transgenic Lines

For these assays, Tg(*EPRE:*EGFP) and Tg(*HSP70:*EGFP) zebrafish larvae were used for the exposures. The Tg(*EPRE:*EGFP) line expresses a fused luciferase (LUC)-green fluorescence protein (EGFP) fusion protein under the regulation of the electrophile response element (EPRE). This EPRE sequence consisted of the mouse *Gsta1* EPRE fused to the minimal promoter from the mouse *mt1* gene. The Tg(*HSP70:*EGFP) line expresses EGFP under the regulation of the *hsp70-4* promoter region. Both transgenic lines have been shown to respond to oxidative stress by increasing the expression of EGFP via the nuclear factor erythroid 2 p45-related factor 2 (Nrf2) pathway in zebrafish [30, 31].

The exposures were carried out as described in Section 2.3, with the addition of 0.2 mM phenylthiourea (PTU) at 24 hpf to prevent the interference of pigmentation on fluorescence imaging. Each experiment consisted of 6–9 embryos per exposure group, and the experiments were repeated twice (*n* = 15–17). At the end of the exposure, animals were individually transferred to a 96-well zebrafish imaging plate (ZF plate, Hashimoto Electronic Industry, Takasucho, Japan) with the addition of 100 *μ*L tricaine (200 mg/L). Images were acquired using a BZ-X700 automated fluorescence microscope (Keyence Corporation of America, Itasca, IL, USA) with 470/40 nm (em) and 525/50 nm (ex) filters. Fluorescence was quantified using ImageJ software and expressed as fold change normalized to the control group.

### 2.11. Determination of Cellular Death

#### 2.11.1. Comet Assay

Genotoxicity was assessed by DNA breaks through the comet assay [50]. After the exposure, twenty larvae per group were selected by simple randomization, carefully homogenized manually with a microtube pestle in 1 mL of Dulbecco's Modified Eagle Medium (DMEN), and centrifuged for 10 minutes at 200 ×g. The pellet containing the isolated cells was collected and diluted in PBS buffer pH 7.4. An aliquot of 10 *μ*L was added to 0.75% low melting agarose and transferred to the slides. The slides were placed at 4°C for 15 minutes and then added in a lysis solution containing 100 mM EDTA, 2.5 M NaCl, 1% Triton X-100, and 10% DMSO (pH 13.0) in the dark at 4°C overnight. Slides were further immersed in a neutralizing solution containing 400 mM Tris at pH 7.4 for 30 minutes. For the unwinding of the DNA, the slides were soaked for 30 minutes in a horizontal electrophoresis tank containing an alkaline buffer (12 g/L NaOH and 0.37 g/L EDTA, pH 13) at 25 V and 300 mA. After the electrophoresis run, the slides were washed in distilled water, fixed with 70% ethanol for 5 minutes, and placed in the refrigerator (4°C) for 1 hour. Finally, slides were stained with SYBR Green for 5 minutes and analyzed by fluorescence microscopy (Olympus 1X71) at 100x magnification, with an exposure of 1044.7 ms, fluorescence at emission (500 nm) and excitation (530 nm) (Olympus U-RFL-T UV light), and an image analysis system (QCapture). The images were analyzed by ImageJ/Open Comet image analysis software.

#### 2.11.2. Apoptosis Analysis *In Vivo* with Acridine Orange Staining

The fluorescent dye Acridine Orange (AO) was used to detect apoptotic cells. After the exposure, fifteen larvae were selected by simple randomization and incubated in 2 mL of 5 *μ*g/mL AO diluted in WMS for 30 min in the dark [51]. Larvae were washed three times in WMS and mounted in slides using 1.5% methylcellulose. Images were obtained by fluorescence microscopy (Olympus 1X71) at 40x magnification and 500 milliseconds of exposure using the filter fluorescein isothiocyanate at emission (530 nm) and excitation (490 nm). Images were analyzed by ImageJ software, and the overall fluorescence in the head area was quantified as a frequency estimate of apoptotic cells.

### 2.12. Mitochondrial Respiration Assays

Mitochondrial bioenergetics were measured *in vitro* by high-resolution respirometry (HRR) using an Oxygraph-2k (O2k, Oroboros Instruments, Innsbruck, Austria). Briefly, one hundred whole body of larval zebrafish were pooled per sample (*n* = 6 per group) and gently homogenized in 70 *μ*L of 5 mM Tris-HCl (pH 7.4) containing 250 mM sucrose and 2 mM EGTA. The homogenate (50 *μ*L) was immediately transferred to 2 mL respiration buffer (3 mM HEPES pH 7.2 containing 115 mM KCl, 10 mM KH_2_PO_4_, 2 mM MgCl_2_, 1 mM EGTA, and 0.2% essentially fatty acid-free BSA 0.2%). All experiments were performed at 28°C using DatLab 4.0 software (Oroboros Inc., Austria), with continuous stirring at 750 rpm.

Using titration protocols [52, 53], we assayed the abilities of a series of substrates and inhibitors to influence mitochondrial function as reflected in differences in respiration states. Succinate or a mixture of glutamate+pyruvate+malate was used as oxidizable substrates in all experiments, as described below. Changes in mitochondrial respiratory chain complexes and respiratory rates were determined.

After signal stabilization, the (i) routine state supported by endogenous substrates was assayed and the (ii) complex I- (CI-) mediated leak (LEAK) respiration was determined using 5 mM pyruvate, 5 mM glutamate, and 1 mM malate in the absence of ADP. (iii) CI-mediated OXPHOS (OXPHOS) was determined with the same substrates but in the presence of ADP (2.5 mM). Based on these values, the respiratory control ratios (RCR = CI_OXPHOS_/CI_LEAK_) can be determined as an indicator of the state of mitochondrial coupling. Next, (iv) the convergent electron flow during the maximal OXPHOS respiration was determined by simultaneously using substrates of the CI (glutamate, pyruvate, and malate) and the complex II (CII, 10 mM succinate) and was named CI+CII_OXPHOS_. The electron transport system (ETS) respiration represents the uncoupled respiration using carbonyl cyanide 4-(trifluoromethoxy)phenylhydrazone (FCCP) as an uncoupler (optimum concentration reached between 0.5 and 1.5 *μ*M), and the (v) CI+CII-mediated ETS respiration (CI+CII_ETS_) was determined using FCCP. (vi) CII-mediated ETS respiration (CII_ETS_) was determined using the CI inhibitor rotenone (0.5 *μ*M). Lastly, (vii) the residual oxygen consumption (ROX) with small contributions from electron leak in the uncoupled state was assayed in the presence of antimycin (2.5 *μ*M), with the inhibition of complex III (CIII), resulting in no mitochondrial respiration.

### 2.13. Citrate Synthase and Lactate Dehydrogenase Activity

Fifty larvae were pooled per sample (*n* = 6 per group) and homogenized in 100 *μ*L of 20 mM potassium phosphate buffer, pH 7.5. Citrate synthase (CS) activity was determined spectrophotometrically in the homogenates according to the method previously described [54]. The enzyme activity in the presence of the acetyl-CoA and oxaloacetate and the resulting CoA-SH was determined at 412 nm and 37°C. The lactate dehydrogenase (LDH) activity was determined in the homogenized sample using a commercial kit (Labtest) following the manufacturer's instructions.

### 2.14. Total Protein Quantification

Protein content was determined using bovine serum albumin (BSA) as standard, according to Bradford [55].

### 2.15. Statistical Analysis

The mean lethal concentration (LC_50_) for 24 h was calculated using the Nonlin fit test. Normality (Kolmogorov-Smirnov) and homogeneity (Bartlett's) tests were applied. The parametric dates are expressed as mean ± standard error (SEM) and were analyzed by one-way ANOVA followed by Tukey post hoc, and nonparametric dates were expressed as median interquartile range, analyzed by Kruskal-Wallis followed by Dunn's post hoc. HRR assays were analyzed by unpaired Students *t*-test. The significance was set at *p* ≤ 0.05.

## 3. Results

### 3.1. Acute PM Exposure Impairs Larval Survival

**Figure 1:**
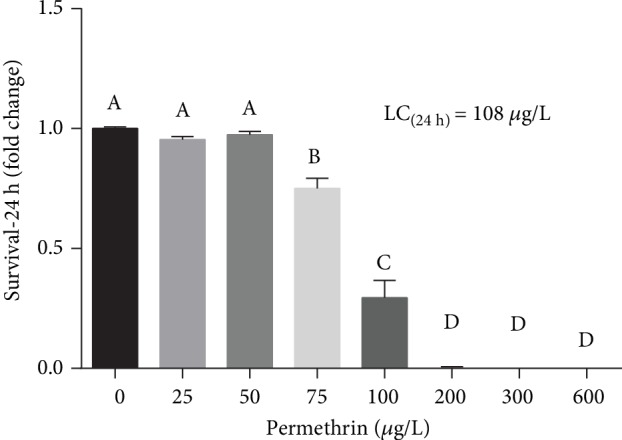
Survival rate (%) of zebrafish larvae after acute exposure (24 h) to different concentrations of permethrin (25, 50, 75, 100, 200, 300, and 600 *μ*g/L) and control group (CTL). Values are expressed as mean ± SEM and were analyzed by one-way ANOVA followed by Tukey as post hoc comparison. Groups not sharing letters are significantly different (*p* < 0.05).

The potential toxicity of PM to zebrafish larvae was initially determined with a 24-hour survival curve. The LC_50_ obtained was 108 *μ*g/L (range, 64.12–182) (Figure 1). The significant effects (F_(7, 104)_ = 150.1, *p* < 0.0001) on a survivor were observed on concentrations higher than 50 *μ*g/L (NOEC). For further experiments, the sublethal concentrations of 25 and 50 *μ*g/L were used.

### 3.2. PM Exposure Modulates Behavioral Functions in Zebrafish Larvae

**Figure 2:**
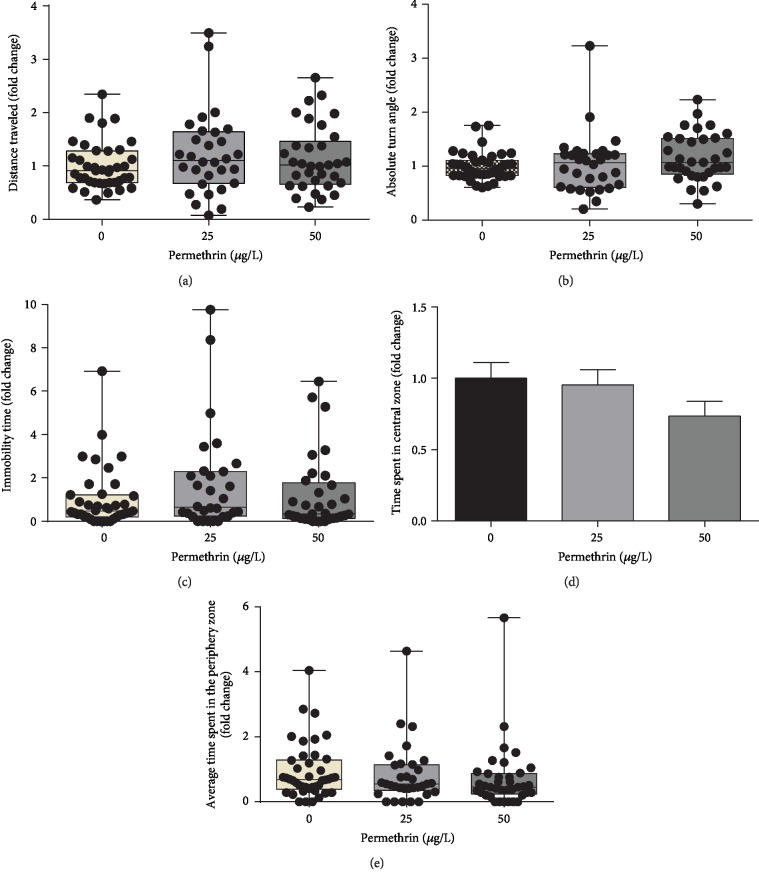
Behavioral assays using the Open Field test to measure the exploratory and motor behaviors in control (CTL) and permethrin (25 and 50 *μ*g/L) exposed zebrafish larvae. (a) Total distance traveled (average for the control group = 0.58 m), (b) absolute turn angle (average for the control group = 16,400°), (c) total immobility time (average for the control group = 27 s), (d) total time spent in the central zone (average for the control group = 92 s), and (e) average time spent in the central zone per visit (average for the control group = 5 s). The parametric dates are expressed as mean ± SEM and were analyzed by one-way ANOVA followed by Tukey post hoc; and nonparametric dates are expressed as median ± interquartile range, analyzed by Kruskal-Wallis followed by Dunn's post hoc. Groups not sharing letters are significantly different (*p* < 0.05).

**Figure 3:**
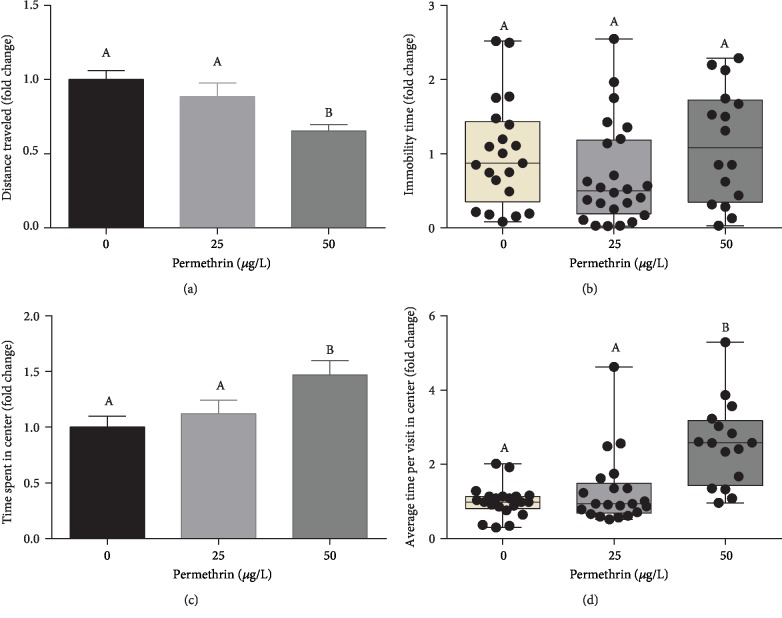
Behavioral assays using the Novel Tank test to measure nonmotor anxiety-like behaviors in control and permethrin (25 and 50 *μ*g/L) exposed zebrafish larvae. (a) Total distance traveled (average for the control group = 0.76 m), (b) total immobility time (average for the control group = 114.3 s), total time spent in the central zone (average for the control group = 84.8 s), and (d) average time spent in the central zone per visit zone (average for the control group = 5 s) were evaluated after exposure time. The parametric dates are expressed as the mean ± SEM and analyzed by one-way ANOVA followed by Tukey as post hoc comparison, and nonparametric dates are expressed as median ± interquartile range, analyzed by Kruskal-Wallis followed by Dunn's multiple comparison test. Groups not sharing letters are significantly different (*p* < 0.05).

In the Open Field test (which has a habituation step), no differences were observed in all evaluated endpoints (Figure 2). In the Novel Tank test (which does not have a habituation step), a significant decrease in distance traveled (*F*_(2, 53)_ = 4.780, *p* = 0.0123) was observed in larvae exposed to 50 *μ*g/L PM (Figure 3(a)). This same exposure group also increased the time spent in the central zone (*F*_(2, 53)_ = 3.192, *p* = 0.0491) (Figure 3(c)) and average time per visit (*F*_(2, 53)_ = 12.56, *p* < 0.0001) (Figure 3(d)). No differences were observed in immobility time (Figure 3(b)).

**Figure 4:**
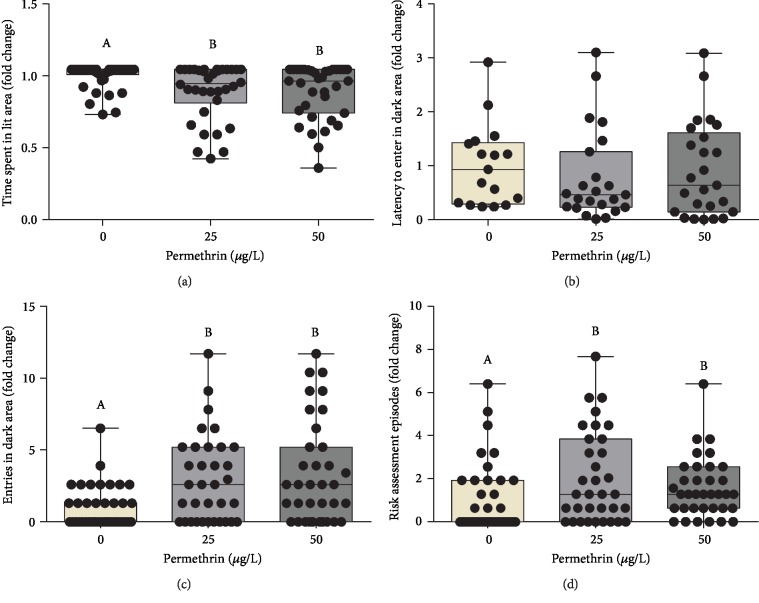
Behavioral assays using the Light-Dark test to measure nonmotor anxiety-like behaviors in control and permethrin (25 and 50 *μ*g/L) exposed zebrafish larvae. (a) Time spent in the lit area traveled (average for the control group = 270 s), (b) latency to enter in the dark area (average for the control group = 67 s), (c) entries to the dark area (average for the control group = 1 entry), and (d) the number of risk assessment episodes (average for the control group = 2 episodes). The dates expressed as median ± interquartile range, analyzed by Kruskal-Wallis followed by Dunn's multiple comparison test. Groups not sharing letters are significantly different (*p* < 0.05).

In the Light-Dark test, it was observed that both PM concentrations decreased the total time spent in the lit area (*F*_(2, 104)_ = 5.659, *p* = 0.0035) (Figure 4(a)) and unchanged the latency to the first enter in the dark area (Figure 4(b)). However, the number of entries in the dark area (*F*_(2, 104)_ = 7.508, *p* = 0.0019) (Figure 4(c)) and episodes of risk assessments (*F*_(2, 104)_ = 3.408, *p* = 0.0064) (Figure 4(d)) were increased in both PM concentrations.

### 3.3. PM Exposure Induces Oxidative Stress

**Figure 5:**
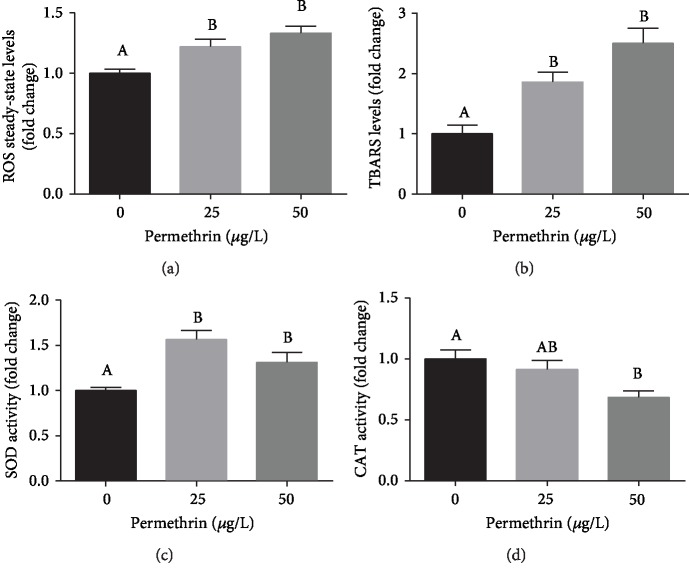
Oxidative stress biomarkers in control (CTL) and permethrin (25 and 50 *μ*g/L) exposed zebrafish larvae. (a) ROS steady-state levels (average for the control group = 0.23 *μ*mol DCF/mg of protein), (b) TBARS levels (average for the control group = 1.6 nmol MDA/mg of protein), (c) CAT activity (average for the control group = 10.6 *μ*mol/min/mg of protein), and (d) SOD activity (average for the control group = 177 U/mg of protein). Values are expressed as mean ± SEM and analyzed by one-way ANOVA followed by Tukey post hoc. Groups not sharing letters are significantly different (*p* < 0.05).

Both concentrations of PM caused a significant increase in ROS steady-state levels (*F*_(2, 30)_ = 8.324, *p* = 0.0013) (Figure 5(a)), TBARS levels (*F*_(2, 27)_ = 13.70, *p* < 0.0001) (Figure 5(b)), and SOD activity (*F*_(2, 20)_ = 12.18, *p* = 0.0003) (Figure 5(c)). The CAT activity (Figure 5(d)) (*F*_(2, 13)_ = 6.503, *p* = 0.0110) decreased only after exposure to 50 *μ*g/L PM.

### 3.4. PM Modulates the Response of the Glutathione Defense System and Proteins Encoded by Nrf2 Target Genes

**Figure 6:**
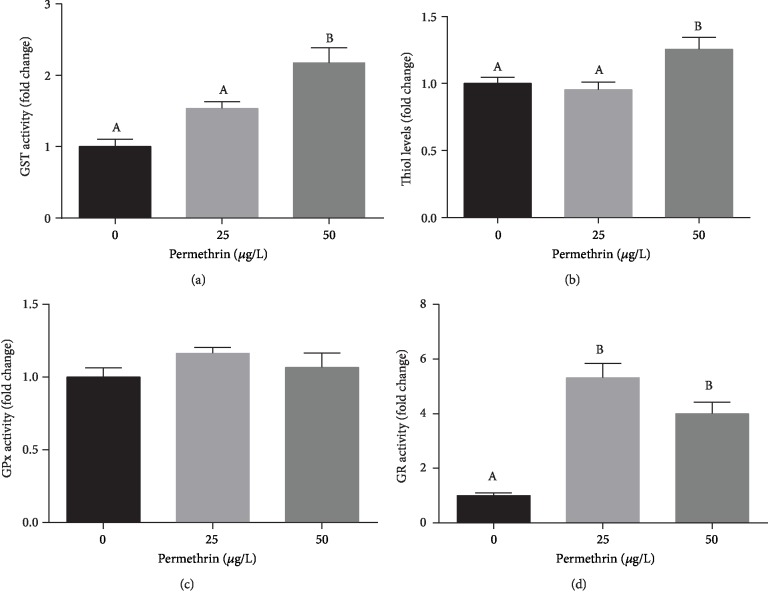
Effects of permethrin exposure (25 and 50 *μ*g/L) in the glutathione defense system in zebrafish larvae. (a) GST activity (average for the control group = 0.16 *μ*mol GS-DNB/min/mg of protein), (b) thiol levels (average for the control group = 330 nmol SH/mg of protein), (c) GPx activity (average for the control group = 16 nmol/min/mg of protein), (d) GR activity (average for the control group = 6.85 nmol/min/mg of protein). Values are expressed as mean ± SEM and analyzed by one-way ANOVA followed by Tukey post hoc. Groups not sharing letters are significantly different (*p* < 0.05).

**Figure 7:**
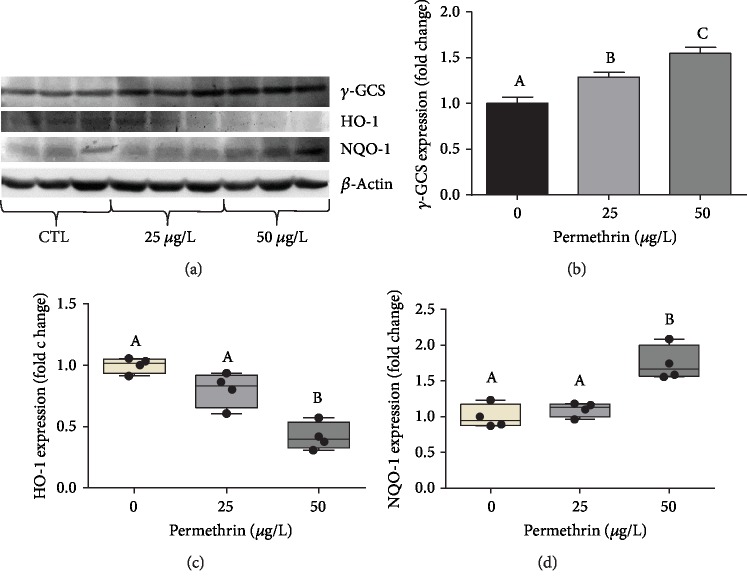
Exposure to permethrin (25 and 50 *μ*g/L) modules the protein levels of Nrf2-target antioxidant enzymes in zebrafish larvae. (a) Representative immunoblots, and densitometric analysis of immunoreactive bands of (b) *γ*-GCS (average for the control group = 0.52*γ*-GSCs/*β*-actin ratio), (c) HO-1 (average for the control group = 1.3 HO-1/*β*-actin ratio), and (d) NQO-1 (average for the control group = 0.63 NQO-1/*β*-actin ratio). The parametric dates are expressed as the mean ± SEM and analyzed by one-way ANOVA followed by Tukey as post hoc comparison, and nonparametric dates are expressed as median ± interquartile range, analyzed by Kruskal-Wallis followed by Dunn's multiple comparison test. Groups not sharing letters are significantly different (*p* < 0.05).

**Figure 8:**
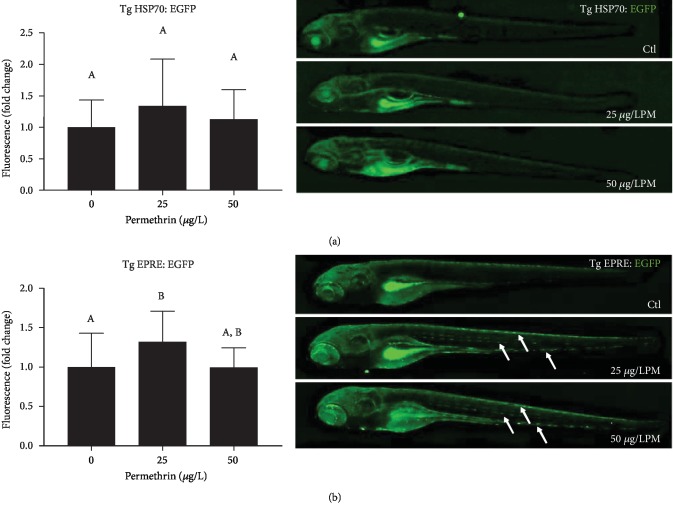
Effects of permethrin exposure (25 and 50 *μ*g/L) in the Nrf2-related antioxidant response in zebrafish larvae. Amplification of the antioxidant system was estimated by fluorescence microscopy using two different zebrafish reporter lines. Expression of EGFP in (a) Tg(*HSP70*:EGFP) and (b) Tg(*EPRE*:LUC-EGFP) in zebrafish larvae. Relative fluorescence levels and representative images are shown for each transgenic construct and exposure group. Arrows indicate the presence of cells expressing high fluorescence in the caudal region of zebrafish larvae. Values are expressed as mean ± SEM and were analyzed by one-way ANOVA followed by Tukey post hoc comparison. Groups not sharing letters are significantly different (*p* < 0.05).

Acute exposure to 50 *μ*g/L PM significantly increased GST activity (*F*_(2, 33)_ = 11.55, *p* = 0.0002) (Figure 6(a)) and the levels of low molecular weight thiols (*F*_(2, 12)_ = 5.911, *p* = 0.0163) (Figure 6(b)). No differences were observed between groups in GPx activity (Figure 6(c)). In addition, GR activity increased in response to both 25 and 50 *μ*g/L PM (*F*_(2, 15)_ = 23.95, *p* < 0.0001) (Figure 6(d)). It was also detected a significant increase in *γ*-GCS (*F*_(2, 15)_ = 18.91, *p* < 0.0001) (Figure 7(b)) and NQO-1 (*F*_(2, 9)_ = 20.63, *p* = 0.0004) (Figure 7(d)) protein levels in larvae exposed to 50 *μ*g/L PM, while a decrease in HO-1 protein levels (*F*_(2, 9)_ = 29.02, *p* = 0.0001) was detected in this same exposure group (Figure 7(b)). This upregulation of the glutathione defense system and Nrf2 targets was further investigated using two transgenic lines with reporter genes responsive to oxidative stress via the Nrf2 pathway. EGFP fluorescence levels were not altered in Tg(HSP70:EGFP) zebrafish (Figure 8(a)), but were increased in Tg(EPRE:EGFP) zebrafish exposed to 25 *μ*g/L PM (Figure 8(b)), particularly in the tail region.

### 3.5. DNA Fragmentation and Cell Death Induced by PM

**Figure 9:**
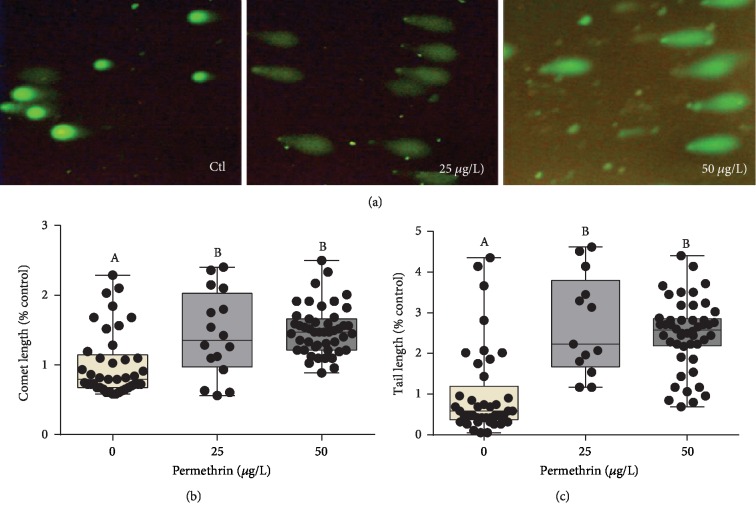
Exposure to permethrin (25 and 50 *μ*g/L) causes genotoxicity in zebrafish larvae. (a) Representative images of single cell gel electrophoresis (comet assay), (b) comet length (average for the control group = 430 microns), and (c) tail length (average for the control group = 195 microns). The dates expressed as median ± interquartile range, analyzed by Kruskal-Wallis followed by Dunn's multiple comparison test. Different letters indicate statistically significant differences (*p* < 0.05).

**Figure 10:**
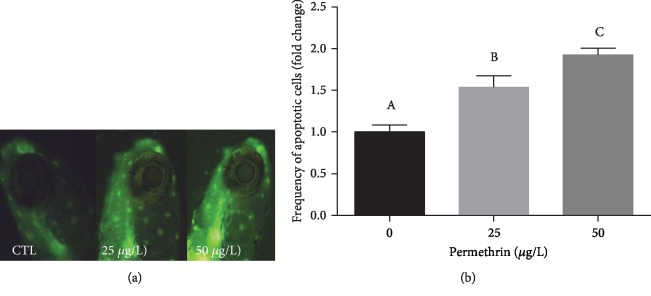
Exposure to permethrin (25 and 50 *μ*g/L) causes cell death by apoptosis in zebrafish larvae. The frequency of apoptotic cells was estimated in the head of zebrafish larvae by measuring the total fluorescence in the area. (a) Representative image of the acridine orange staining and fluorescence imaging, indicating the presence of apoptotic cells (white arrows), and (b) the respective quantification of acridine orange relative fluorescence. Values are expressed as mean ± SEM and were analyzed by one-way ANOVA followed by Tukey post hoc comparison. Different letters indicate statistically significant differences (*p* < 0.05).

PM exposure induced DNA damage and apoptosis as evaluated by the comet assay and AO stain, respectively. Both PM exposure groups had a significant increase in the lengths of the whole comet (*F*_(2, 101)_ = 20.94, *p* < 0.0001) (Figure 9(b)) and the tail (*F*_(2, 101)_ = 30.04, *p* < 0.0001) (Figure 9(c)). This same pattern was observed in the AO test, with a substantial increase in the relative fluorescence in the head of the larvae (*F*_(2, 31)_ = 20.31, *p* < 0.0001) (Figure 10(b)), demonstrating increased in the apoptotic cell death induced by acute exposure to both PM concentrations.

### 3.6. PM Exposure Decreases All States of Mitochondrial Respiration

**Figure 11:**
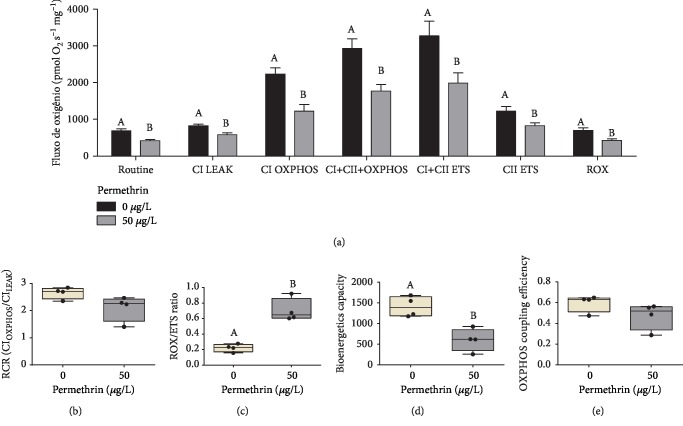
Effects of permethrin exposure (50 *μ*g/L) in mitochondrial function of zebrafish larvae. (a) The analyzed mitochondrial function by HRR is presented with the abbreviation(s) of the complex(es) involved followed by the state of respiration measured: basal metabolic rate (routine), +glutamate, pyruvate and malate (CI LEAK), +ADP (CI OXPHOS), +succinate (CI+CII CII_OXPHOS_), +FCCP (CI+CII ETS), +rotenone (CII ETS), +antimycin A (ROX, residual oxygen consumption). Average for the basal control group = 600 pmol O_2_/s/mg. (b) Respiratory control ratio (RCR) for complex I (RCR = CI_OXPHOS_/CI_LEAK_). (c) Residual oxygen consumption relative to the maximum oxygen consumption capacity (ROX/ETS). (d) Analysis of bioenergetics capacity (CI_LEAK_-CIoxphos). (e) Analysis of oxidative phosphorylation coupling efficiency (CI_LEAK_/CI_OXIPHOS_). Values are expressed as mean ± SEM and analyzed by one-way ANOVA followed by Tukey post hoc or expressed as median ± interquartile range and analyzed by Kruskal-Wallis followed by Dunn's post hoc. Groups not sharing letters indicate statistical differences (*p* < 0.05) between the exposure groups at each state of respiration.

To investigate the effects of PM at the mitochondrial level, we verified the mitochondrial bioenergetics function using HRR (Figure 11(a)). Only the control and PM 50 *μ*g/L groups were evaluated in this assay. All states of mitochondrial respiration were significantly decreased after PM exposure: basal (*t*0.05; 6 = 4.082, *p* = 0.0065), CI_LEAK_ (*t*0.05; 6 = 3.452, *p* = 0.0136), CI_OXPHOS_ (*t*0.05; 6 = 4.042, *p* = 0.0068), CI+CII_OXPHOS_ (*t*0.05; 6 = 6.674, *p* = 0.0104), CI+CII_EST_ (*t*0.05; 6 = 3.865, *p* = 0.0083), CII_ETS_ (*t*0.05; 6 = 2.821, *p* = 0.0303), and Ama (*t*0.05; 6 = 3.383, *p* = 0.0148). Thereby, PM-related effects were detected in the mitochondrial CI and CII activity both in the dissipative components due to proton leak, proton slip, cation cycling, and electron leak states (LEAK) or the ADP-activated state (OXPHOS). Analysis of the total maximum oxygen flux consumption (CI+CII_ETS_) or the CII-dependent maximum oxygen flux consumption (CII_ETS_) by FCCP-dependent decoupling of the ETS also showed significant decrease in oxygen flux consumption by PM exposure. The addition of antimycin A resulting in residual oxygen consumption (ROX) returned the oxygen flux consumption to values close to basal.

### 3.7. PM Exposure Changes Other Mitochondrial Parameters, including Mitochondrial Flux Control Ratio, Bioenergetics Capacity, and Substrate Control Ratio

Additional analyses of mitochondrial metabolism were carried out to further investigate the effects of PM at the mitochondrial level. The respiratory control ratio (RCR) is an indicator of mitochondrial coupling and was used to evaluate mitochondrial functionality; the PM exposure did not change the RCR values (Figure 11(b)). The PM exposure was able to significant increase the magnitude of residual oxygen consumption (nonmitochondrial respiration) relative to the maximum oxygen consumption capacity (FMAX) (*t*0.05; 6 = 6.208, *p* = 0.0008), which was determined by the ROX/ETS ratio (Figure 11(c)). In addition, the exposure to PM decreased the mitochondrial bioenergetics capacity (*t*0.05; 6 = 4.374, *p* = 0.0047) (Figure 11(d)), which was quantified by subtracting the ADP-induced CI_OXPHOS_ values from the CI_LEAK_. Lastly, PM exposure did not alter the OXPHOS coupling efficiency (Figure 11(e)), which was estimated as a result of the CI_LEAK_/CI_OXPHOS_ ratio.

**Figure 12:**
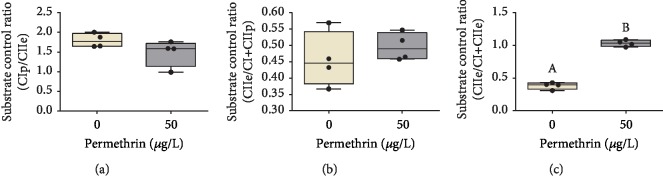
Analysis of the substrate control ratio (SCR) of zebrafish larvae exposed to permethrin 50 *μ*g/L. (a) CI_OXPHOS_/CII_ETS_ ratio, (b) CII_ETS_/CI+CII_OXPHOS_ ratio, and (c) CII_ETS_/CI+CII_ETS_ ratio. The dates expressed as median ± interquartile range, analyzed by Kruskal-Wallis followed by Dunn's multiple comparison test. Different letters indicate statistically significant differences (*p* < 0.05).

In order to determine the PM exposure effects on mitochondrial respiratory control, the quantified substrate control ratio (SCR) was determined. The PM exposure was not able to alter the CI_OXPHOS_/CII_ETS_ ratio (Figure 12(a)). However, when the contribution of CII in the convergent electron flow through the Q-junction was evaluated in mitochondrial respiratory control, we noticed no differences in CII_EST_/CI+CII_OXPHOS_ (Figure 12(b)) and significant increase of CII_EST_/CI+CII_ETS_ (*t*0.05; 6 = 18.58, *p* < 0.0001) (Figure 12(c)) induced by exposure to PM.

### 3.8. PM Exposure Alters the Enzymatic Metabolism of Glucose

**Figure 13:**
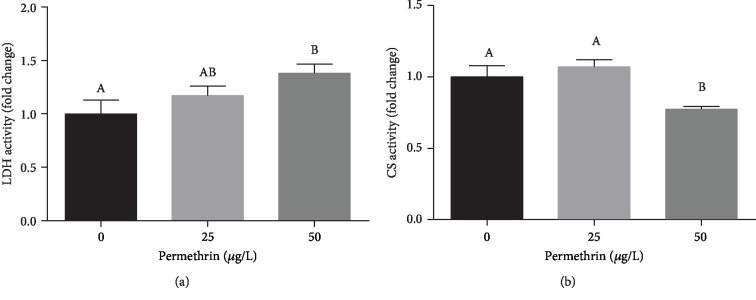
Effects of permethrin exposure (25 and 50 *μ*g/L) in biomarkers of glucose metabolism in zebrafish larvae. (a) Lactate dehydrogenase activity (average for the control group = 750 U/L) and (b) citrate synthase activity (average for the control group = 0.036 nmol/min/mg). Values are expressed as mean ± SEM and were analyzed by one-way ANOVA followed by Tukey post hoc comparison. Groups not sharing letters are significantly different (*p* < 0.05).

Zebrafish larvae exposed to 50 *μ*g/L of PM had significantly increased LDH activity (*F*_(2, 17)_ = 3.555, *p* = 0.0423) (Figure 13(a)) and decreased CS activity (*F*_(2, 18)_ = 7.816, *p* = 0.0036) (Figure 13(b)) when compared to the control group.

## 4. Discussion

Pyrethroids are considered safer in relation to other pesticides, mostly due to their low toxicity to mammals and low environmental impact. Approximately one out of ten pesticides commonly used at homes and gardens has pyrethroids as active ingredients [56]. As a result, preschool children have been found to be potentially exposed to PM from several sources and through several routes in their daily environments [9, 57, 58]. These facts have attracted attention to the effects of pesticides in developing organisms, which are under intense and controlled cell proliferation, differentiation, and apoptosis [3]. These events are closely associated with strict regulation of cellular redox homeostasis [14, 15, 59], which can be potentially altered by the redox alterations triggered in response to SP exposures, including PM [5]. In this study, we analyzed the toxic effects of acute PM in a developmental animal model and demonstrated significant toxic effects at the behavioral, mitochondrial, and molecular level, which appears to be related to redox alterations and consecutive cell death.

Our results demonstrated a significant decrease in larval survival (6 dpf) after exposure for 24 hours to PM (≥75 *μ*g/L). The estimated LC_50_ was 108 *μ*g/L, one-third of the LC_50_ showed by other researchers [18, 34] in zebrafish exposure to PM from 3 to 144 hpf (100–800 *μ*g/L of PM). Yang et al. [18] reported a great increase in the mortality rate, morphological alterations, and behavior changes in 6 dpf larvae exposed to higher levels of PM (≥300 *μ*g/L), a similar developmental stage to the one used in the current study. Therefore, it is possible that these later developmental stages can be more susceptible to PM toxicity, once the gill innervation and respiration start between 5 and 7 dpf [60]. Fish are the most sensitive vertebrate organisms to SP due to their chemical lipophilic property while having a high absorption rate through the gills [61].

Changes in normal behavioral patterns in zebrafish larvae were the first signs of toxicity to sublethal PM exposure. This behavioral profile is an effective method to characterize the effects of different compounds on swimming activity of fish, predicting the potential action of a compound in the central nervous system [62]. Such types of neurodevelopmental deficits usually include a broad spectrum of disorders and dysfunctions, namely, developmental delays, behavioral problems, and deficits in gross or fine motor skills [12]. In the present study, no differences were observed in all endpoints analyzed in the Open Field test, demonstrating that sublethal PM exposure does not impair overall larval locomotor activity. The mode of action of pyrethroids, generally, involves binding to voltage-gated sodium channels causing prolonged opening and disruption of the channel (Soderlund, 2012), impairing motor activity (Wolansky et al., 2009). This mechanism is conserved among vertebrates; however, the sublethal PM concentrations tested in this work unable to impair the larvae motor activity. Similar behavior results were demonstrated by Awoyemi et al. [63], where larvae exposed (5 hpf–5 dpf) to PM concentrations of 0.1–1000 *μ*g/L did not show changes in morphological and locomotor behaviors.

We detected, for the first time, that PM exposure caused alterations in nonmotor behavioral patterns, such as decreased defensive behaviors in the Novel Tank and Light-Dark tests linked to thigmotaxis and scototaxis. Despite the exploratory patterns not been altered in the Open Field test, PM-exposed larvae submitted to the stress of a novel environment (Novel Tank test) had decreased distance traveled. This result may be attributed to increase anxiety, which in this test is accompanied by decreased exploration [62, 64]. However, the Novel Tank test adapted to zebrafish larvae is possible to evaluate the thigmotaxis, where animals that are engaged in thigmotaxic behavior strongly avoid the central area, presenting a preference to stay or to move in the peripheral area of a novel environment [65]. This behavior is evolutionarily conserved in a wide range of fish species [37, 65–69] and can be modulated by anxiogenic drugs [65, 67]. The exposure to PM may have decreased this anxiety-like behavior in zebrafish larvae, altering the normal preference of the periphery in favor of the central area, based on the increased total time spent and the average time spent per visit in the central area. The results from the Light-Dark test corroborate with this hypothesis. PM exposure changed the normal preference of the lit area, observed by an increase in the number of entries into the dark area, which was linked to increase in the number of risk assessment episodes. This test assesses the natural behavior of zebrafish larvae to avoid dark zones (scototaxis) [41, 70] and also suggest modulation in the anxiety-like phenotype in zebrafish larvae exposed to PM.

The concentrations tested in this work may be considered environmentally relevant, once the concentrations found in the environmental water column = 0.05–811 *μ*g/L [18, 63]. Thereby, it is possible that behavioral changes observed in our laboratorial conditions can reflect environmental exposure conditions to PM. In the natural environmental, fear and anxiety function to avert situations that may be harmful to health or overall well-being, such as avoiding predators. The molecular mechanisms involved in physiological responses to danger are conserved among vertebrates and are essential for the survival and maintenance of the species [37]. Thereby, the environmental exposure to PM may impair the normal behavior and survival of nontarget species and, consequently, reduce environmental quality and influence essential ecosystem functioning by reducing species diversity.

The brain can be particularly susceptible to oxidative stress due to its high oxygen consumption and lower antioxidant activity [71, 72]. Excessive production and/or insufficient degradation of ROS can cause oxidative damage in astrocytes and/or neurons, followed by acute brain injury, neurodegenerative diseases, and altered motor and nonmotor functions [73]. Besides that, the increase in ROS steady-state levels in response to toxins can cause adverse effects through the further production of peroxides and other free radicals. In this study, we demonstrated the increase of ROS steady-state levels as well as the increase in MDA in larvae exposed to 50 *μ*g/L PM, possibly as a result of such elevated ROS levels. MDA is a marker of lipid peroxidation, the most abundant individual aldehyde resulting from this oxidation event, which can be easily quantified [74, 75]. Lipid peroxidation is commonly used as a biomarker of oxidative stress in fish and contributes to impaired cellular function under oxidative conditions [76].

To evaluate the response to the possible oxidative effects mediated by the increased ROS, enzymes of the antioxidant system were analyzed. CAT and SOD are important antioxidant enzymes which play pivotal roles in the first antioxidative defense line of organisms [77]. In the review published by Wang et al. [5], it is discussed that the antioxidant enzymes affected by PM exposure are presented in a gender-, dose-, time-, tissue-, or enantioselective-dependent manner. We observed an inhibitory effect of PM in CAT activity in whole larvae exposed to 50 *μ*g/L PM. On the other hand, SOD activity increased in both exposure groups. Moderate oxidative stress may be upregulated the antioxidant enzymes; however, under high ROS levels, their activities can be decreased [78]. SOD dismutates O_2_^•−^ to H_2_O_2_, which is further metabolized by GPx and CAT [79]. The O_2_^•−^ can also react with nitric oxide (^•^NO) leading to the formation of peroxynitrite (ONOO^−^), while H_2_O_2_ can react with myeloperoxidases to produce hypochlorous acid or be reduced to hydroxyl radical (OH^•−^) through Fenton reactions [80]. Therefore, the proper regulation of both O_2_^•−^ and H_2_O_2_ needs to be accurately regulated by the cell.

Upregulation of glutathione-related defenses was also detected after PM exposure, probably in response to increasing ROS formation. Increased thiol levels (including GSH) and GST activity can help to mediate the phase II biotransformation of unsaturated aldehydes, which are generated during lipid peroxidation and can then be excreted through a GS-X pump transporter [81, 82]. While the biotransformation of pyrethroids in fish involves oxidation and hydrolysis reactions and conjugation with glucuronic acid or sulfate [83], GSH has been associated with protection against PM-induced DNA damage in rats [84] and GST knockdown increases the susceptibility of ticks to PM [85]. It remains unclear if such GSH/GST response has a direct effect in the metabolism of PM, but its role as an effective cellular protection mechanism against ROS oxidative reactions is well known and could also benefit from the increased GR activity after PM exposure.

Awoyemi et al. [63] showed that larvae zebrafish exposed to environmental relevant concentrations of PM upregulated Nrf2 expression [63]. This pathway is activated under oxidative and electrophilic stress conditions, when Nrf2 is released from its sequester (Kelch-like ECH-associated protein 1 (Keap1)) and translocated to the nucleus to increase the expression of antioxidant and phase II biotransformation genes [86, 87]. The increased SOD, GR, and GST activities in parallel to the high thiol levels and increased *γ*-GCS and NQO-1 protein levels after PM exposure indicate the possible activation of this pathway. This was further investigated using two zebrafish transgenic lines with reporter genes regulated under the Nrf2-related antioxidant response element/electrophile response element (ARE/EPRE): Tg(*EPRE*:LUC-EGFP) and Tg(*HSP70*:EGFP). Both of them have been used before to characterize the response of the Nrf2 pathway, and the data from the Tg(*EPRE*:LUC-EGFP) indicate an overall increase in the activity of the Nrf2 pathway after PM exposure. Apparently, PM exposure causes an increase in ROS levels in zebrafish larvae, which is followed by the upregulation of key antioxidant molecules via a classic regulator of the antioxidant and phase II biotransformation system.

Our results also demonstrated that treatment with acute sublethal doses of PM caused genotoxicity, as well as an increase in apoptotic cells in the head of zebrafish larvae. It is known that cells exposed to PM have increased oxidation rates of pyrimidine and purine bases, which can be associated with cell death [88, 89]. Besides that, the increase in ROS levels and lipid peroxidation could be linked to the activation of cell death pathways [90]. Thereby, following oxidative stress, cell death can occur via apoptotic or necrotic mechanisms [91]. It is important to note that NQO-1 can also act as a prooxidant enzyme, producing intermediates that are capable of alkylating nucleophilic sites including DNA [59, 92]. It is unclear if the higher protein levels of NQO-1 after PM exposure are associated with this mechanism of DNA damage, which could contribute to the cell death detected in zebrafish larvae.

The oxidative stress induced by PM exposure in larvae zebrafish has the potential to alter various bioenergetics parameters, such as oxygen consumption, the activity of the electron transport chain, or mitochondrial membrane potential, and all these effects can impair the rate of mitochondrial ATP production [93]. Besides that, the normal mitochondrial function includes an unavoidable leak of proton and electron within mitochondria, which generates O_2_^•−^. The unbalance on these side reactions can cause a series of deleterious effects [22]. Furthermore, damage to this organelle, for example, by environmental contaminants, can play both a direct role and an indirect role in the ROS generation and can induce a drastic impairment in cellular bioenergetics metabolism [23]. In this context, considering the critical role of mitochondria in toxicological processes, we investigated whether the mitochondrial function was a target of PM in zebrafish larvae. Our results indicated that PM exposure impairs bioenergetics capacity and increases the residual oxygen consumption, but does not impair mitochondrial efficiency and oxidative phosphorylation. However, we demonstrate that exposure to PM impaired also the mitochondrial function through the decrease in all states of mitochondrial respiration. We demonstrated that PM can impair redox homeostasis, which is known to potentially oxidize mitochondrial protein thiol groups, triggering mitochondrial transition permeability and increasing ROS production rates [52, 94, 95]. In addition, SCR results indicate that PM exposure changes the mitochondrial bioenergetics metabolism of NADH oxidation, indicating a compensatory response of the CII respiration in relation to the convergent pathway, which is able to trigger the accentuated superoxide generation in mitochondria [96].

The prooxidative condition could trigger the release of iron from the iron-sulfur center present in CI and II, which may exacerbate ROS generation via Fenton-like mechanisms [97]. Thereby, the impairment effects of PM on mitochondrial function can be associated with the decrease in HO-1 expression observed in larvae exposed to 50 *μ*g/L PM. The HO-1 is the rate-limiting enzyme of heme degradation, catalyzing heme degradation and generating ferrous iron, carbon monoxide, and biliverdin, the latter two of which have anti-inflammatory and antioxidant properties [98, 99]. In oxidative stress conditions, HO-1 may translocate to the mitochondria and the generated carbon monoxide may play an important role in mitochondrial biogenesis, suggesting an intimate link between HO-1 and mitochondrial function during stress [100, 101]. Additionally, the toxicity of both hydrogen and lipid peroxides is greatly enhanced by reacting with heme or heme-containing proteins that generate strong prooxidant ferryl forms of heme [102]. Thereby, it is possible that the lower protein levels of HO-1 after PM exposure may affect mitochondrial biogenesis or decrease the rate of heme degradation and both of these scenarios can be toxic to the mitochondria.

Effects on mitochondrial function can shift the cell metabolism towards an inefficient way of producing ATP at the cost high fluxes of anabolic pathways. We showed an increase in LDH (glucose metabolism) and decrease in CS activity (citric acid cycle) in PM-exposed larvae. These alterations can be related to the Warburg effect [103], a mechanism that occurs in tumors and other cells under proliferation or development, where glycolysis is the major energetic pathway even in the presence of oxygen. The NADH generated from glucose is reoxidized to NAD^+^, reducing pyruvate to lactate and completing the aerobic glycolysis. Despite this scenario showed a different situation than the one proposed in the Warburg effect, a similar mechanism can have been adopted as an attempt to maintain ATP levels by glycolysis even in the presence of oxygen. PM exposure decreased mitochondrial oxygen flux consumption and potentially decreased activity of the citric acid cycle (CS activity) and increased glucose anabolism (LDH activity); thus, it is possible that glycolysis is been used to provide the extra fuel necessary for physiological brain function through the generation of ATP. Glucose is the foundation for neuronal and nonneuronal cellular maintenance, as well as the generation of neurotransmitters [104]. It is unclear if such changes observed in the regulation of glucose metabolism by PM exposure in zebrafish larvae have a side effect, as glucose consumption is expected to be higher and this is a critical molecule for brain physiology, which may lead changes in anxiety-like behavior observed.

In conclusion, we showed that acute and sublethal PM exposure modulates different anxiety-like behavior patterns in zebrafish larvae. Additionally, we also demonstrate that PM enhances enzymatic antioxidant defenses, with focus on the glutathione-related system; however, this upregulation was not enough to prevent the increase in ROS levels and oxidative effects such as lipid peroxidation, DNA damage, and cell death. It is possible that unbalance in the redox state of the cells can be either a cause or consequence of changes in mitochondrial function and glucose metabolism. We also emphasize that behavioral alterations, as well as the changes in redox parameters and mitochondrial function in larval zebrafish, may serve as important tools to better understand the molecular mechanisms induced by acute exposure to PM in developing organisms.

## Data Availability

The data used to support the findings of this study are available from the corresponding author upon request.
